# NF-кB increases LPS-mediated procalcitonin production in human hepatocytes

**DOI:** 10.1038/s41598-018-27302-7

**Published:** 2018-06-11

**Authors:** Yongfeng Bai, Jun Lu, Ying Cheng, Feng Zhang, Xueyu Fan, Yuanyuan Weng, Jin Zhu

**Affiliations:** Core Facility, Department of Clinical Laboratory, Quzhou People’s Hospital, Quzhou, Zhejiang, China

## Abstract

For years, procalcitonin (PCT) has been employed as a diagnostic biomarker for the severity of sepsis and septic shock, as well as for guiding the application of antibiotics. However, the molecular/cellular basis for the regulation of PCT production is not fully understood. In this study, we identified the signalling pathway by which the expression of PCT was induced by lipopolysaccharide in human hepatocytes at the mRNA and protein levels. This expression was dependent on nuclear transcription factor κB (NF-κB), as indicated by a NF-κB binding site (nt −53 to −44) found in the PCT promoter region. We also showed that microRNA-513b (miR-513b) was also able to bind to the 3′-untranslated region (UTR) of the PCT promoter sequence. Meanwhile, the activation of NF-κB down-regulated the expression of miR-513b. In conclusion, we suggest that NF-κB is capable of enhancing the expression of PCT by either directly activating the transcription of the PCT gene or indirectly modulating the expression of its regulatory component, miR-513b. Our results indicate a molecular mechanism responsible for the regulation of PCT production.

## Introduction

Procalcitonin (PCT), the precursor of the hormone calcitonin, comprises 116 amino acids and is present in minute quantities under healthy conditions^1^. The level of PCT has been observed to increase significantly with bacterial infection and correlate to the severity of the infection^2^. Therefore, serum PCT has been considered a powerful biomarker for the diagnosis of bacterial infection. This marker has also been employed successfully to guide the administration of antibiotics^3^. Since the discovery of its association with sepsis in the 1990s, many studies on PCT and its clinical applications have been conducted^4^. However, the molecular details of the regulation of PCT production in relation to bacterial infection remain partially understood.

Nuclear transcription factor κB (NF-κB), an important master transcription factor, is involved in the regulation of numerous components of the host immune response^5^. NF-κB is naturally located in the cytoplasm bound to its inhibitory proteins, known as inhibitors of NF-κB (IκBs). Upon stimulation by various activators, one of which is lipopolysaccharide (LPS), the IκB complex is degraded to release NF-κB protein^6^, and the latter is then allowed to translocate to the nucleus and attach to specific binding site(s) on target genes. As a master transcriptional regulator, NF-κB is responsible for modulating the production of many cellular components, including antimicrobial peptides, cytokines, chemokines, stress-response proteins and anti-apoptotic proteins^7^.

MicroRNAs (miRs) are a group of small, non-coding, single-stranded RNAs that regulate gene expression by base pairing with the untranslated regions (UTRs) of their target genes. Those small molecules are considered the tools for fine tuning the stability of mRNA and consequently affecting the translational outcomes^8^. The miR-513 subfamily belongs to the miR-506–514 cluster. Evidence shows that different members of the miR-513 subfamily (miR-513a/b/c) lead to functional divergences and that miR-513b can affect male sexual maturation by negatively regulating the development stage-related function of DR1^9^. Recent studies have indicated that miR-513b inhibits cell proliferation in testicular embryonal carcinoma^10^ and gastric cancer^11^. Furthermore, miR-513b is decreased in cholangiocytes following *Cryptosporidium parvum* infection or LPS stimulation and is associated with B7-H1 expression^12,13^. Collectively, these observations suggest that miR-513b plays an important role in regulating the host inflammatory response.

In this study, we conclude that NF-κB signalling activation is necessary for PCT expression in human hepatocytes. On the one hand, NF-κB can directly regulate the production of PCT by binding to its promoter. On the other hand, NF-κB is a negative regulator of miR-513b, which is an inhibitor of PCT expression. Taken together, our results not only indicate that NF-κB is crucial for PCT production in bacterial infection but also provide a detailed mechanism of PCT generation in hepatocytes.

## Results

### LPS stimulates PCT expression in hepatocytes

**Figure 1:**
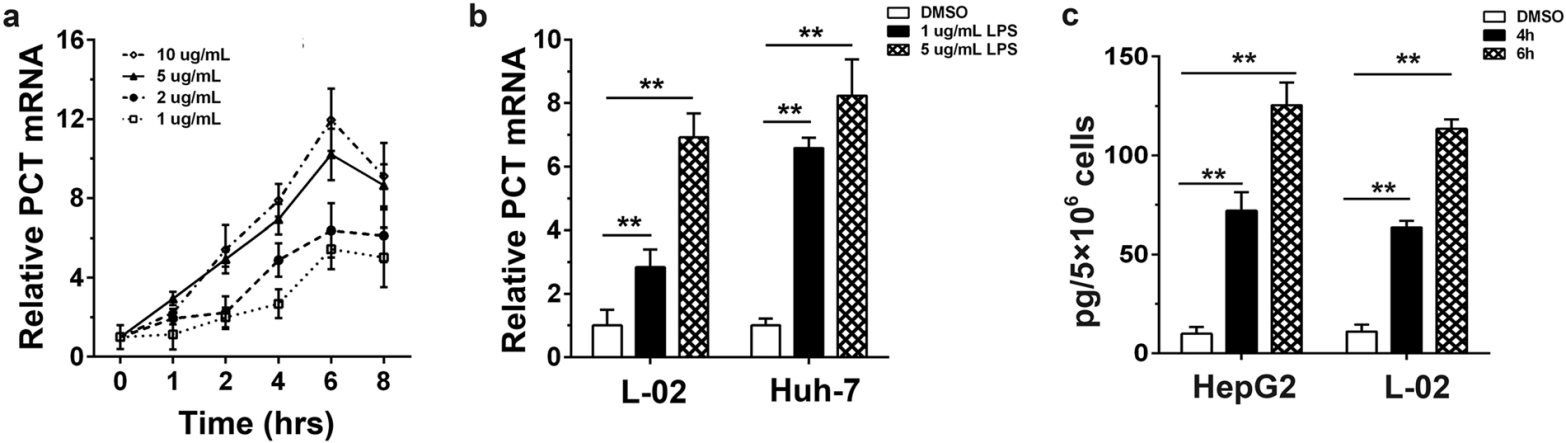
Stimulation of hepatocytes with LPS elevates PCT expression. **(a)** HepG2 cells were treated with different concentrations of LPS (1, 2, 5, 10 μg/mL) for the indicated time. Quantitative real-time PCR was performed to detect the amount of PCT mRNA. The relative expression of PCT was normalized to the β-actin content. The mean value at 0 hr was used as a standard, and the fold changes were calculated with the ΔΔCt method. Data are represented as the mean ± SD of three experimental replicates. **(b)** L-02 and Huh-7 cells were treated with LPS (1, 5 μg/mL) or DMSO solvent as a control for 6 hrs, and the amount of PCT mRNA was assessed by qPCR. The mean value after treatment with DMSO was used as a standard, and the fold changes were calculated with the ΔΔCt method. Data are represented as the mean ± SD of three experimental replicates. **(c)** HepG2 and L-02 cells were treated with LPS (5 μg/mL) or DMSO solvent as a control for 4 or 6 hr, and PCT protein secretion in the culture medium was measured. Data are represented as the mean ± SD of three experimental replicates. ^**^p < 0.01.

To verify the effect of LPS on PCT expression, we initially measured PCT expression upon stimulation with different concentrations of LPS in HepG2 cells. The level of PCT expression was examined by real-time PCR. The results showed that the level of PCT expression increased upon LPS treatment in a dose-dependent manner. LPS treatment triggered a much more dramatic change in PCT expression when increasing the concentration from 1 μg/mL to 5 μg/mL than from 5 μg/mL to 10 μg/mL (Fig. 1a). We chose 5 μg/mL as the LPS concentration for further study. The temporal patterns of PCT production in HepG2 cells at various LPS concentrations were similar. The expression of PCT rapidly increased by 2 hr, reached its highest levels by 6 hr, and started to decrease by 8 hr after LPS treatment (Fig. 1a). Similarly, significant PCT expression was also induced after stimulation with LPS for the indicated time periods in L-02 and Huh-7 cells (Fig. 1b). Meanwhile, the content of PCT in the cell culture supernatant also increased, accompanied by elevated PCT mRNA levels (Fig. 1c).

### NF-кB activation is required for LPS-induced PCT expression

**Figure 2:**
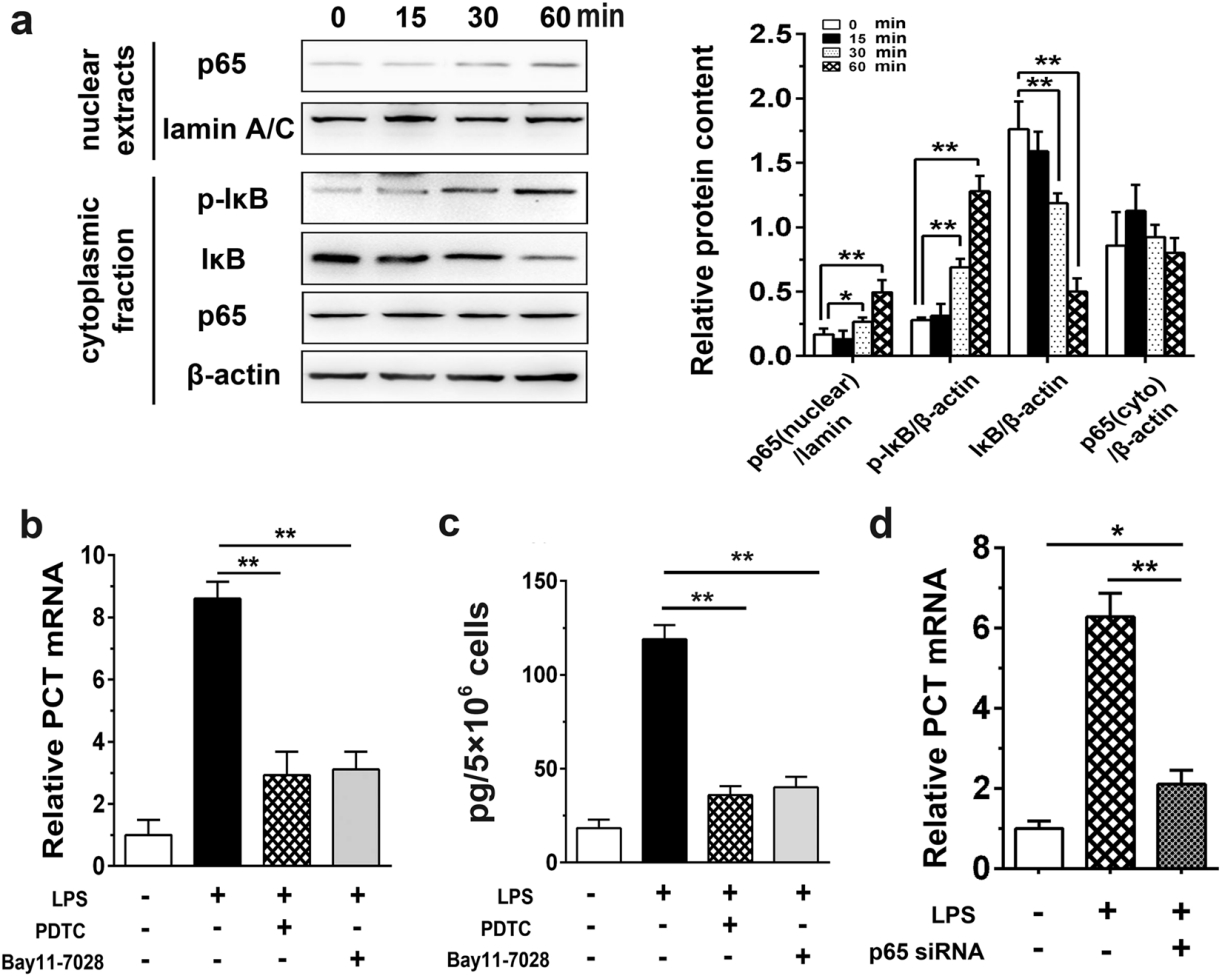
LPS induces PCT expression in HepG2 hepatocytes through NF-κB activation. **(a)** HepG2 cells were stimulated with LPS (5 μg/mL) for the indicated time. Cytoplasmic and nuclear extracts were assessed by Western blotting using specific antibodies against IκBα, p-IκBα and NF-κB p65, as well as β-actin and lamin A/C as loading controls for the cytoplasmic and nuclear fractions, respectively. Left, representative data of three independent experiments. Right, quantitative analysis of the band intensities of three independent experiments. Full-length blots are presented in Supplementary Fig. S1. **(b**,**c)** HepG2 cells were pretreated with PDTC (30 μg/mL) or Bay11-7082 (3 μg/mL) for 1 hr and stimulated with LPS (5 μg/mL) for 6 hr. The amount of intracellular PCT mRNA **(b)** and PCT protein in the supernatant **(c)** was detected, respectively. Data are represented as the mean ± SD of three experimental replicates. **(d)** HepG2 cells transiently transfected with NF-κB p65 siRNA were treated with LPS (5 μg/mL) for 6 hr. The level of PCT mRNA expression was detected by qPCR. Data are represented as the mean ± SD of three experimental replicates. ^*^p < 0.05; ^**^p < 0.01.

NF-κB is one of the most important regulators of proinflammatory gene expression upon LPS stimulation^7^. Activation of the NF-κB pathway upon LPS treatment was indicated by substantially increased IκBα phosphorylation and degradation as well as NF-κB p65 accumulation in the nuclear fraction in human hepatocytes (Fig. 2a). To explore the role of the NF-κB pathway in LPS-induced PCT expression, we pretreated HepG2 cells with an NF-κB inhibitor, either PDTC or Bay11-7082. The NF-κB inhibitors substantially attenuated the PCT mRNA and protein levels in LPS-stimulated HepG2 cells (Fig. 2b,c). Furthermore, we depleted NF-κB p65 with siRNA in HepG2 cells followed by exposure to LPS. As shown in Fig. 2d, NF-κB p65 inhibition greatly reduced LPS-induced PCT expression at the mRNA level.

### Identification of NF-κB binding sites in PCT promoter

**Figure 3:**
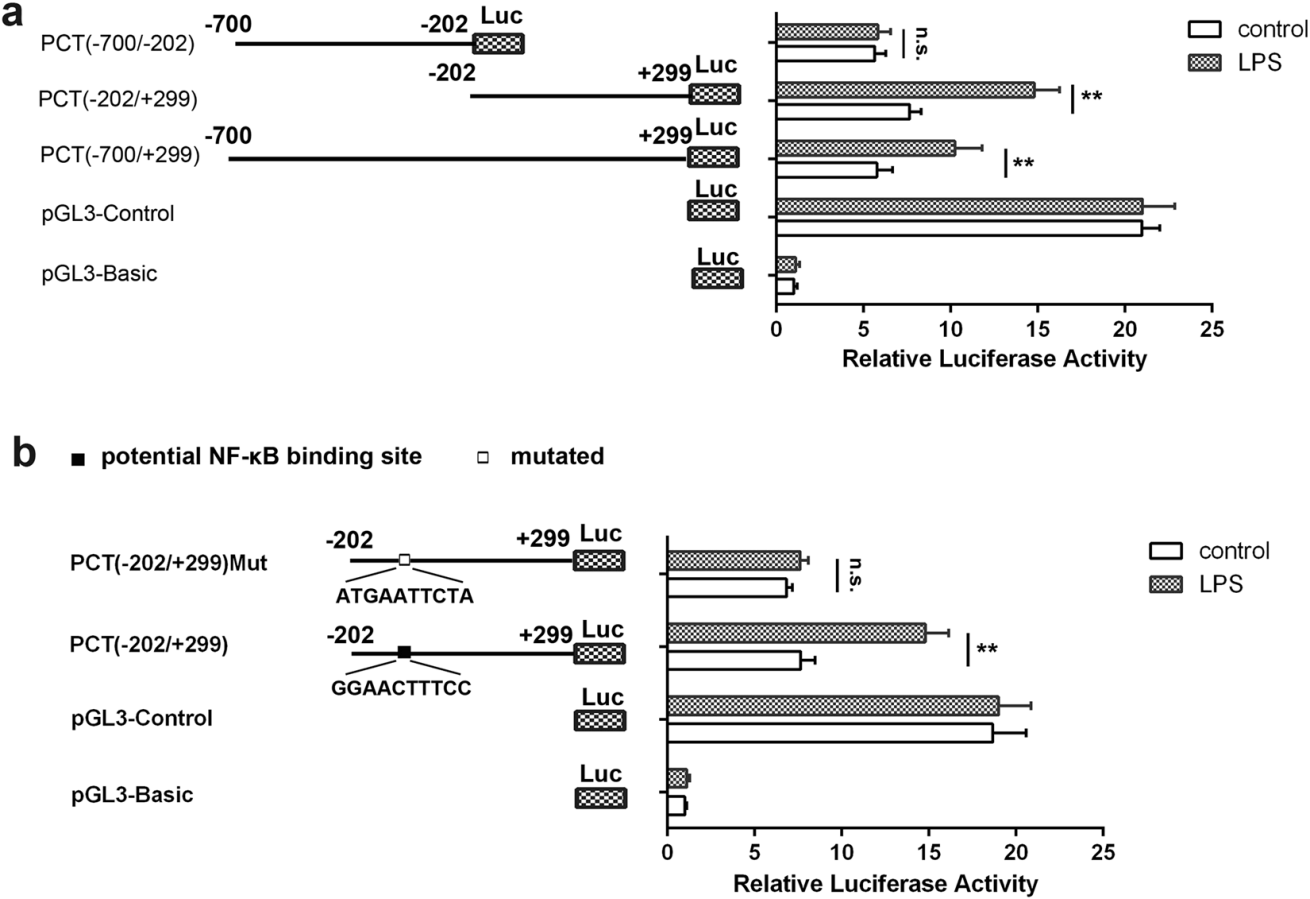
Identification of NF-κB binding sites in the PCT promoter. **(a)** HepG2 was transfected with luciferase reporter plasmids containing different lengths of PCT promoter fragments together with pTK-Renilla luciferase plasmid. Twenty-four hours later, the cells were treated with 5 μg/mL LPS for 6 hr, and luciferase activity was measured and normalized to Renilla luciferase activity. Data are represented as the mean ± SD of six experimental replicates. **(b)** HepG2 cells were transfected with PCT (−202/+299) or PCT (−202/+299) Mut luciferase reporter together with pTK-Renilla luciferase plasmid. Twenty-four hours later, the cells were stimulated with 5 µg/ml LPS for 6 hr, and luciferase activity was measured and normalized to Renilla luciferase activity. Data are represented as the mean ± SD of six experimental replicates. ^**^p < 0.01; n.s., no significance.

Since NF-κB is a transcription factor, we considered whether it promoted PCT expression through direct binding to the PCT gene promoter. Luciferase reporter gene vectors with promoter segments of different lengths were constructed and transiently transfected into HepG2 cells. As shown in Fig. 3a, LPS stimulation significantly increased the luciferase activity with the PCT (−700/+299) and PCT (−202/+299) reporters, respectively. Conversely, LPS could not induce luciferase activity with the PCT (−700/−202) reporter, indicating that the cis elements responsible for LPS-induced PCT expression are located in the PCT promoter region from nt −202 to +299.

To identify NF-κB binding sites, the PCT promoter fragment from nt −202 to +299 was analysed in the TRANSFAC 6.0 database. The putative NF-κB binding sequence GGAACTTTCC (nt −53 to −44) was identified. To confirm the function of this NF-κB binding site in LPS-induced PCT expression, we mutated it in the PCT (−202/+299) reporter to generate the PCT (−202/+299) Mut reporter. Mutation of the NF-κB binding site significantly attenuated LPS-induced luciferase activation compared to the control (wild type) reporter (Fig. 3b). Together, these data indicate that NF-κB enhanced PCT expression upon LPS treatment through direct binding to the PCT promoter.

### miR-513b modulates PCT mRNA expression in hepatocytes

**Figure 4:**
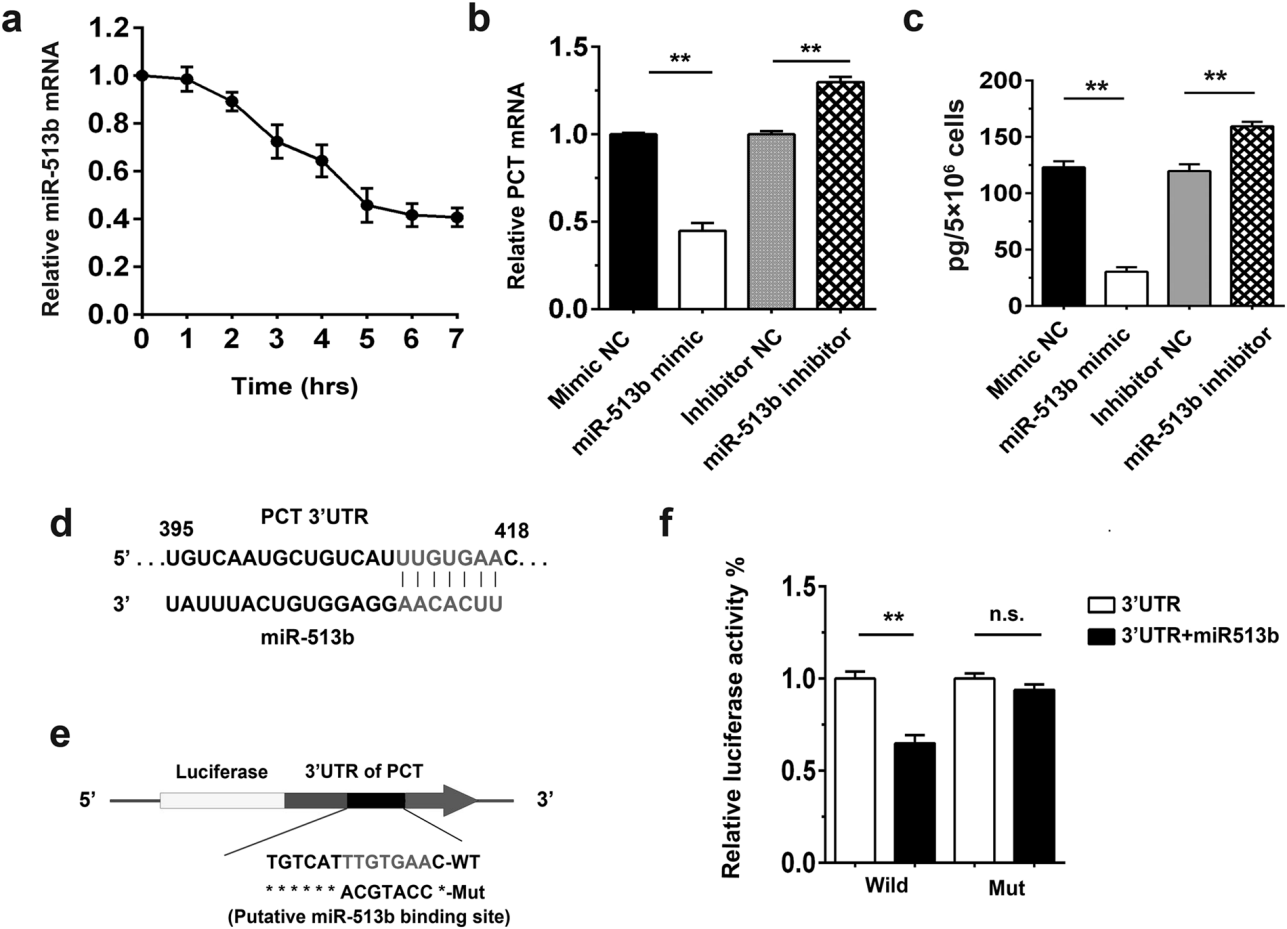
miR-513b inhibits PCT production. **(a)** HepG2 cells were treated with 5 μg/mL LPS for the indicated time. Quantitative real-time PCR was performed to detect the kinetics of miR-513b expression after LPS treatment. The relative expression of miR-513b was normalized to the U6 content. The mean values at 0 hr were used as a standard. Data are represented as the mean ± SD of three experimental replicates. **(b,c)** HepG2 cells were transfected with 100 nM NC mimic or miR-513b mimic or with 150 nM inhibitor negative control (inhibitor NC) or miR-513b inhibitor for 24 hr followed by stimulation with 5 µg/ml LPS for 6 hr. The level of intracellular PCT mRNA **(b)** and PCT protein in the supernatant **(c)** was detected. Data are represented as the mean ± SD of three experimental replicates. **(d)** Putative binding sites for miR-513b were predicted in the 3′-UTR of PCT mRNA. **(e)** The complementary miR-513b binding site in the PCT 3′-UTR was inserted downstream of a luciferase reporter on the psiCHECK-2 plasmid. A control plasmid with the mutant 3′-UTR sequence was also generated. WT, wild type. **(f)** Relative luciferase activity in 293 T cells transfected with PCT-3′-UTR or PCT-3′-UTR Mut and either miR-513b mimic or NC mimic. The normalized luciferase activity in the control group was used to determine the relative luciferase activity. Data are represented as the mean ± SD of six experimental replicates. ^**^p < 0.01; n.s., no significance.

Using bioinformatics analysis, we found that PCT is a potential target of miR-513b. To determine whether miR-513b is involved in PCT expression, we examined the expression of miR-513b upon LPS treatment. Hepatocytes were treated with LPS for the indicated time. The results show that the miR-513b mRNA level decreased gradually after LPS treatment (Fig. 4a). As mentioned above, PCT expression increased in HepG2 cells upon LPS stimulation (Fig. 1a), which was inversely correlated with the change in miR-513b expression. We hypothesized that the correlation between PCT and miR-513b indicated a regulatory association in PCT expression after LPS stimulation in HepG2 cells. To confirm our hypothesis, we enhanced or inhibited miR-513b expression using miR-513b mimics or miR-513b inhibitors, respectively. Ectopic miR-513b expression significantly suppressed PCT mRNA production after LPS stimulation, while miR-513b inhibition enhanced PCT expression compared to the control cells (Fig. 4b). Meanwhile, similar results were also obtained at the protein level (Fig. 4c).

To verify whether PCT is a direct target of miR-513b, we generated several luciferase constructs that contained the 3′-UTR of PCT with either the wild type or mutated putative miR-513b binding site (TTGTGAA to ACGTACC) (Fig. 4d,e). We then transfected HepG2 cells with each reporter construct along with miR-513b or negative control (NC) mimics. The miR-513b mimics significantly decreased the PCT-3′-UTR luciferase activity. In the controls, no difference in luciferase activity was found in HepG2 cells transfected with PCT-3′-UTR Mut compared with the NC mimic (Fig. 4f). These observations suggest that miR-513b binds to the putative binding site in the PCT mRNA 3′-UTR, leading to the inhibition of PCT expression.

### NF-κB activation inhibits miR-513b expression upon LPS stimulation

**Figure 5:**
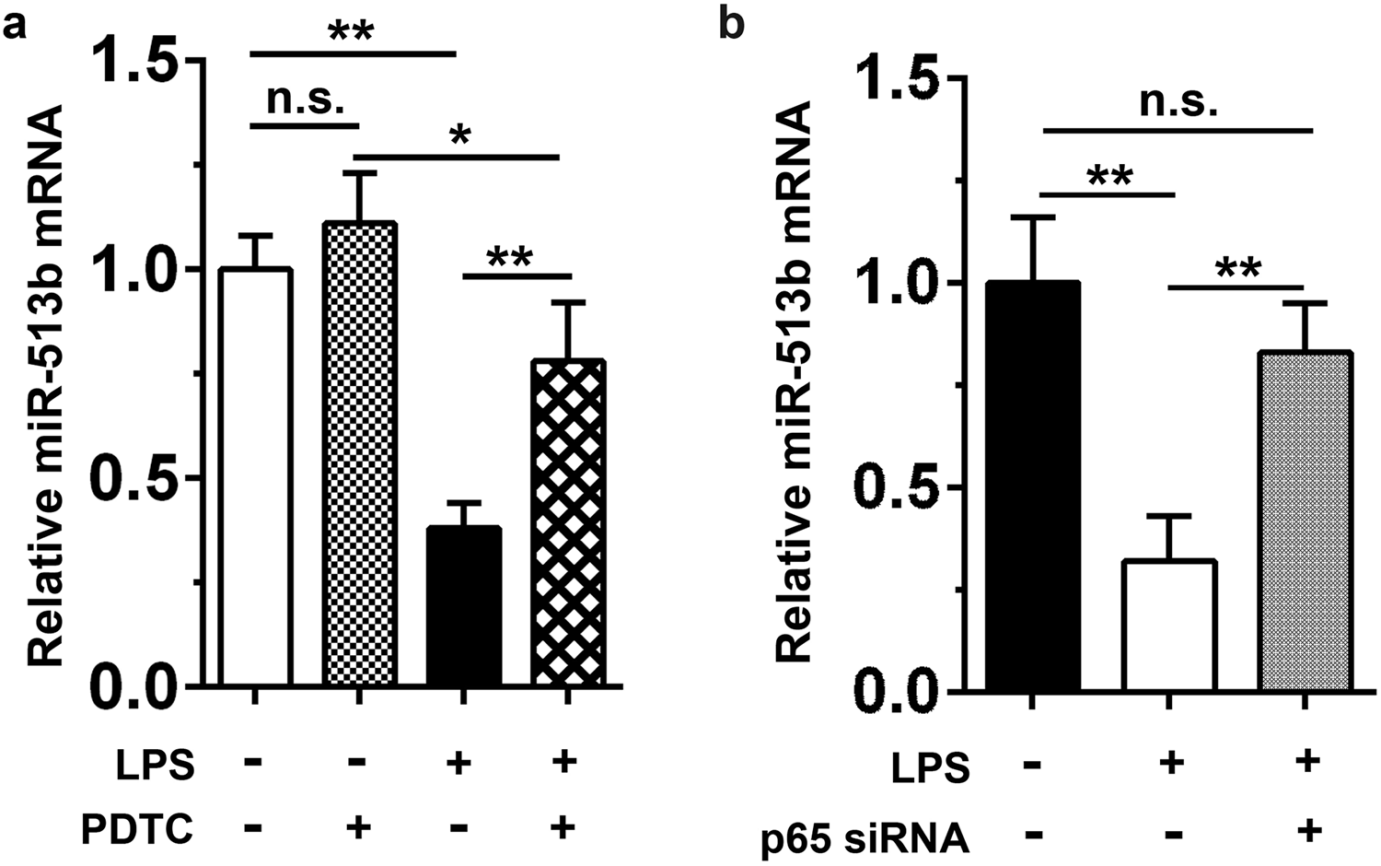
NF-κB regulates PCT expression through miR-513b. (**a)** HepG2 cells were pretreated with PDTC (30 μg/mL) or DMSO solvent as a control for 1 hr and stimulated with LPS (5 μg/mL) for 6 hr. The amount of miR-513b mRNA was detected using qPCR. Data are presented as the mean ± SD of six experimental replicates. **(b)** siRNA specifically targeting human NF-κB p65 subunit (50 nM) was transiently transfected into HepG2 cells followed by stimulation with LPS (5 μg/mL) for 6 hr. The level of miR-513b mRNA was detected using qPCR. Data are represented as the mean ± SD of three experimental replicates. ^*^p < 0.05; ^**^p < 0.01; n.s., no significance.

Numerous investigations have supported the role of miRs in immune responses and inflammation, which are also known to be regulated by NF-κB^14^. To test whether the NF-κB pathway is involved in regulating miR-513b expression, we pretreated HepG2 cells with the NF-κB inhibitor PDTC, followed by exposure to LPS for 6 hr. As shown in Fig. 5a, PDTC treatment relieved the inhibitory effect of miR-513b upon LPS stimulation. In addition, the knockdown of NF-κB p65 by specific siRNA significantly alleviated the reduction in miR-513b compared to the control upon LPS stimulation (Fig. 5b). These results demonstrated that NF-κB signalling is required for the inhibition of miR-513b expression upon LPS stimulation.

## Discussion

PCT originates from the calcitonin-related polypeptide alpha (CALCA) gene on chromosome 11^15^. It is produced ubiquitously in response to endotoxins or to mediators released in response to bacterial infections. As such, it has become the mostly widely used biomarker in the management of sepsis around the world. However, the details of the mechanisms regulating PCT expression are not fully understood. In previous research, our team found that miR-125b regulates PCT production in monocytes by targeting STAT3^16^. However, the release of PCT by monocytes is a transient process and is not a major source of PCT in the circulation^17^. In septic hamsters, PCT mRNA was significantly increased by various types of parenchymal cells (e.g., hepatocytes), as shown by *in situ* hybridization^18^. Moreover, PCT could be detected in the supernatant after stimulating liver slices, which are composed of various cell types, with TNF-α and IL-6^19^. However, the question of whether parenchymal hepatocytes are capable of synthesizing and secreting PCT remains. Here, we show that LPS treatment strongly induced PCT synthesis at the mRNA and protein levels in human hepatocytes, which suggests that infection-mediated PCT expression is not limited to one type of cell^20^, as exemplified by parenchymal hepatocytes.

NF-κB is clearly one of the most important regulators of proinflammatory gene expression. NF-κB regulates the synthesis of many cytokines, such as TNF-α, IL-1β, IL-6, and IL-8^5^. However, whether it is involved in the regulation of PCT expression is unknown. For the first time, we show that the transcription of PCT, whose promoter region had previously been identified^15^, is under the positive regulation of NF-κB. NF-κB belongs to the category of “rapid-acting” primary transcription factors, which are present in cells in an inactive state and do not require new protein synthesis to become activated. This characteristic may explain the rapid PCT synthesis after LPS treatment.

miRNAs have been indicated to play a key role in the regulation of normal immunity and the inflammation response. For instance, it has been shown that lipomannan from virulent mycobacterium tuberculosis blocks TNF biosynthesis through the induction of miR-125b, which binds to the 3′-UTR region of TNF mRNA and destabilizes the transcript^21^. miR-155 overexpression led to significantly reduced IL-8 production induced by *H*. *pylori* infection^22^. Although miRNAs are important for immunity, the role of miRNAs in the regulation of PCT production remains unexplored. In this study, our luciferase reporter assay revealed that PCT is a direct target downstream of miR-513b and that miR-513b overexpression can negatively regulate the generation of PCT. Our findings indicate that miR-513b plays a key role in the immune response to bacterial infection through PCT production.

The NF-κB signalling pathway regulates the transcription of many miRNAs. Lam *et al*. suggested that NF-κB signalling may be implicated in the induction of BIC/miR-155 expression in lymphoma cells^23^. Recently, it was reported that the Notch and NF-κB signalling pathways regulate the miR-223/FBXW7 axis in T-cell acute lymphoblastic leukaemia^24^. Using NF-κB inhibitors and siRNAs, we found that the inhibition of miR-513b expression upon LPS treatment is dependent on the activation of NF-κB.

**Figure 6:**
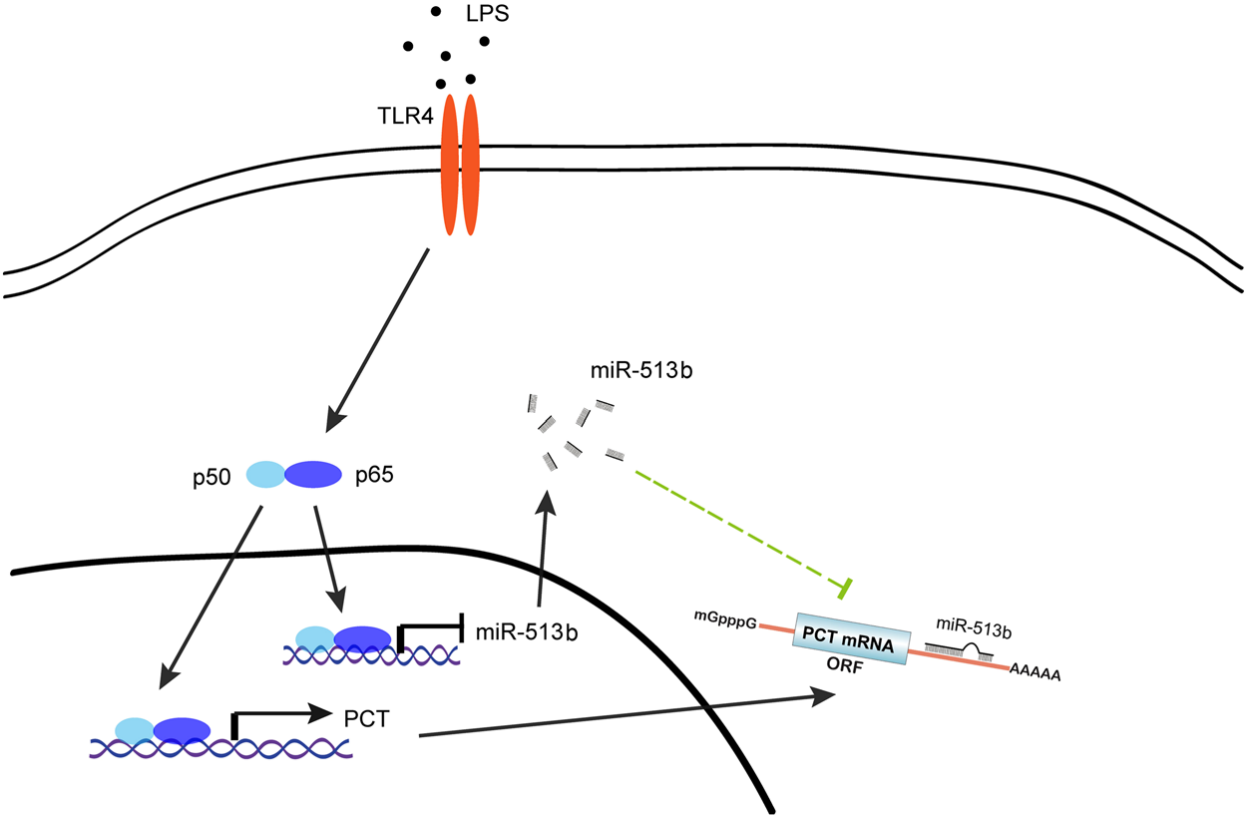
A proposed model of LPS-induced PCT expression mediated by NF-κB and miR-513b.

In conclusion, we have demonstrated that the transcription factor NF-κB is involved in the regulation of PCT transcription in LPS-stimulated hepatocytes. On the one hand, NF-κB can directly bind to the promoter region of the CALCA gene to induce PCT production; on the other hand, LPS suppresses the expression of miR-513b by activating NF-κB to upregulate PCT expression (Fig. 6). Our study offers new insight into the immune response activity conferred by miR-513b and the potential mechanisms of PCT production.

## Methods

### Cell culture and chemicals

HepG2, L-02, Huh-7 and 293T cells were routinely maintained in DMEM (Life Technologies) supplemented with 10% foetal bovine serum (Life Technologies) and 100 U/ml penicillin in a humidified incubator containing 5% CO_2_ at 37 °C. NF-κB inhibitors PDTC (ammonium pyrrolidine dithiocarbamate) and BAY11-7082 were purchased from Beyotime (Beijing, China). LPS from the *E*. *coli* strain O111:B4 was purchased from Sigma-Aldrich (St. Louis, MO).

### PCT protein secretion

PCT protein in the supernatant was analysed after 100-fold concentration using vacuum freeze-drying. Elecsys PCT (Roche) was run on a Cobase 601 analyser, with electrochemiluminescence immunoassay (ECLIA) for protein determination. According to the manufacturers’ information, the detection limit of Elecsys PCT assays is ≤0.02 ng/mL, and the functional sensitivity of Elecsys is 0.06 ng/mL. Basal PCT production was below the limit of quantification in unstimulated hepatic cells *in vitro*.

### RNA preparation and quantitative real-time PCR

Total cellular RNA was isolated using TRNzol universal reagent according to the manufacturer’s protocol (Tiangen Biotech, Beijing, China)). miRNA was reverse-transcribed using M-MTV117 reverse transcriptase (Takara, Otsu, Japan) with miR-513b-RT and U6-RT primers. cDNA was synthesized using 1 μg of total RNA digested with DNase I and random primer and then subjected to SYBR green (Takara, Otsu, Japan)-based real-time PCR. The amplification primers for miR-513b, U6, PCT and β-actin are listed in Table 1. The cycling parameters were 94 °C for 90 s and then 40 cycles of 98 °C (10 s), 58 °C (25 s), and 72 °C (25 s), followed by a melting curve analysis. All reactions were performed with four technical replicates and repeated independently three times. The cycle threshold values were normalized to the expression of U6 small nuclear RNA or the housekeeping gene β-actin.

**Table 1:** Sequences of Oligonucleotides Used in This Article.

Name	Sequence (5′ to 3′)
U6-RT	CGCTTCACGAATTTGCGTGTCAT
miR-513b-RT	GTCGTATCCAGTGCAGGGTCCGAGGTATTCGCACTGGATACGACATAAAT
U6-F	GCTTCGGCAGCACATATACTAAAAT
U6-R	CGCTTCACGAATTTGCGTGTCAT
miR-513b-F	GCGCTTCACAAGGAGGTGT
miR-513b-R	GTGCAGGGTCCGAGGTATTC
β-actin-F	AGAGCTACGAGCTGCCTGAC
β-actin-R	AGCACTGTGTTGGCGTACAG
PCT-F	GGAGAGCAGCCCAGCAGACCC
PCT-R	GTTGGCATTCTGGGGCATGCTAA
PCT (−700/+299)-F	CGGGGTACCGGAAACTCTTGTAGATCCCTGCCTA
PCT (−700/+299)-R	CCCAAGCTTGCGAGCCGGGGGATTGAGACTGT
PCT (−202/+299)-F	CGGGGTACCGCCCCCACCATCCCCCACCATTT
PCT (−202/+299)-R	CCCAAGCTTGCGAGCCGGGGGATTGAGACTGT
PCT (−700/−202)-F	CGGGGTACCGGAAACTCTTGTAGATCCCTGCCTA
PCT (−700/−202)-R	CCCAAGCTTAAATGGTGGGGGATGGTGGGGGC
NC mimic-sense	UUCUCCGAACGUGUCACGUTT
NC mimic-antisense	ACGUGACACGUUCGGAGAATT
miR-513b-mimic-sense	UUCACAAGGAGGUGUCAUUUAU
miR-513b-mimic-antisense	AAAUGACACCUCCUUGUGAAUU
NC inhibitor	CAGUACUUUUGUGUAGUACAA
miR-513b-inhibitor	AUAAAUGACACCUCCUUGUGAA
PCT-3′-UTR-F	CTACTCGAGGCAGCTGAATGACTCAAGAAGGTCA
PCT-3′-UTR-R	CGGGCGGCCGCTGAAGGGTATAGAAAATACATTTTT

### Analysis of nuclear and cytoplasmic proteins by Western blot

Cells were harvested and washed with PBS, followed by centrifugation at 300 × g for 10 min. The cell pellet was lysed with NE-PER nuclear and cytoplasmic extraction reagent according to the manufacturer’s protocol (Thermo Fisher Scientific). The protein concentration was determined by Lowry’s method using bovine serum albumin (BSA) as a standard. Monoclonal antibodies to NF-κB p65, IκBα, phospho-IκBα, and β-actin were purchased from Cell Signalling, Inc. Monoclonal lamin A/C antibody was purchased from Thermo Fisher Scientific. Western blotting was carried out as previously described. Monoclonal sheep anti-mouse IgG or donkey anti-rabbit IgG horseradish peroxidase-conjugated secondary antibodies were used at a ratio of 1:3000. Proteins were assayed by a chemiluminescence (ECL) reagent using a commercial kit (Tanon Biotechnology, Shanghai, China).

### Construction of plasmids

The PCT (NM_001741) promoter fragments nt −700 to +299, −202 to +299, and −700 to −202 were amplified by PCR using human whole-blood genomic DNA as a template and inserted into the KpnI and HindIII sites of the luciferase reporter plasmid pGL3-basic vector (Promega), yielding the reporter constructs PCT (−700/+299), PCT (−202/+299), and PCT (−700/−202), respectively. The primer sequences are listed in Table 1. In the NF-κB point mutants, the potential NF-κB binding site (nt-53 to −44) in the PCT (−202/+299) reporter was replaced with a non-functional sequence using a Site-Directed Mutagenesis Kit (Yeasen, Shanghai, China), yielding the reporter construct PCT (−202/+299) Mut. All constructs were confirmed by DNA sequencing.

The 3′-UTR of the PCT gene containing the miR-513b binding site predicted by TargetScan (http://www.targetscan.org/) was inserted into the XhoI and NotI sites of the psiCHECK-2 dual-luciferase reporter vector and designated PCT-3′-UTR (454 bp). The plasmid PCT-3′-UTR Mut containing the mutated binding site of miRNA-513b in the 3′-UTR generated by PCR-based site-directed mutagenesis was also constructed. All constructs generated were verified by DNA sequencing.

### Oligonucleotides

The miR-513b mimic and miR-513b inhibitor, as well as the scrambled NC, were chemically synthesized and purified by GENEWIZ (Suzhou, China). The miR-513b mimic is a double-stranded RNA mimic mature endogenous miR-513b, and the miR-513b inhibitor is a single-stranded 2-O-methyl-modified oligo ribonucleotide fragment antisense to miR-513b. The oligonucleotide sequences are listed in Table 1. Human NF-κB/p65 siRNA and NC siRNA were obtained from Santa Cruz Biotechnology, Inc. (Santa Cruz, CA).

### Luciferase reporter gene assay

To identify the NF-κB binding site in the PCT promoter, HepG2 cells (1 × 10^5^) were plated in 24-well dishes and cotransfected with 400 ng of the pGL3-reporter plasmid and 20 ng of the pRL-TK-Renilla luciferase plasmid using Lipofectamine 3000 transfection reagent (Invitrogen, Carlsbad, CA). After 48 hr, the cells were treated with LPS for 6 hr. Luciferase activity levels were measured with a Dual-Luciferase Reporter Gene Assay Kit (Beyotime, Beijing, China) according to the manufacturer’s instructions. Firefly and Renilla luciferase activity levels were determined, and values were normalized to the relevant vector control.

To analyse miR-513b targets, 293 T cells cultured in 24-well plates were transiently cotransfected with 500 ng of luciferase vector PCT-3′-UTR or PCT-3′-UTR Mut and either 100 nM miR-513b mimics or scrambled NC using Lipofectamine 3000. The cells were lysed and the luciferase activity was measured using a Dual-Luciferase Assay after 48 hr.

### Transfection with siRNA

HepG2 cells were seeded and transfected in 24-well plates. After seeding HepG2 cells at 1 × 10^5^ cells/well, the cells were reverse transfected with 50 nM siRNA or NC siRNA using Lipofectamine 3000 according to the manufacturer’s protocol.

### Statistical analysis

All data are shown as the mean ± SD (standard deviation). Statistical tests were performed using an unpaired T test (Figs 3 and 4) and two-way ANOVA (Figs 1,2 and 5) with GraphPad PRISM 6 software. P < 0.05 was considered significant.

## Electronic supplementary material

Supplementary Information
