# Supplementary material for: NF-кB increases LPS-mediated procalcitonin production in human hepatocytes
Source: Sci Rep. 2018 Jun 11;8:8913. doi: 10.1038/s41598-018-27302-7 (PMC5995812; doi:10.1038/s41598-018-27302-7)

Supplementary Information file

NF-κB increases LPS-mediated procalcitonin production in human hepatocytes

Yongfeng Bai[1], Jun Lu[1], Ying Cheng[1], Feng Zhang[1], Xueyu Fan[1], Yuanyuan Weng[1], Jin Zhu[1*]

[1]Core Facility, Department of Clinical Laboratory, Quzhou People's Hospital, Quzhou, Zhejiang, China.

Running title: NF-κB regulates procalcitonin production

[*]**Correspondence and requests for materials should be addressed to:** Jin Zhu (email: qzhosp@163.com), Core Facility, Department of Clinical Laboratory, Quzhou People's Hospital, Quzhou, Zhejiang, China.

Supplemental Figure S1: Full western blot of Figure 2a

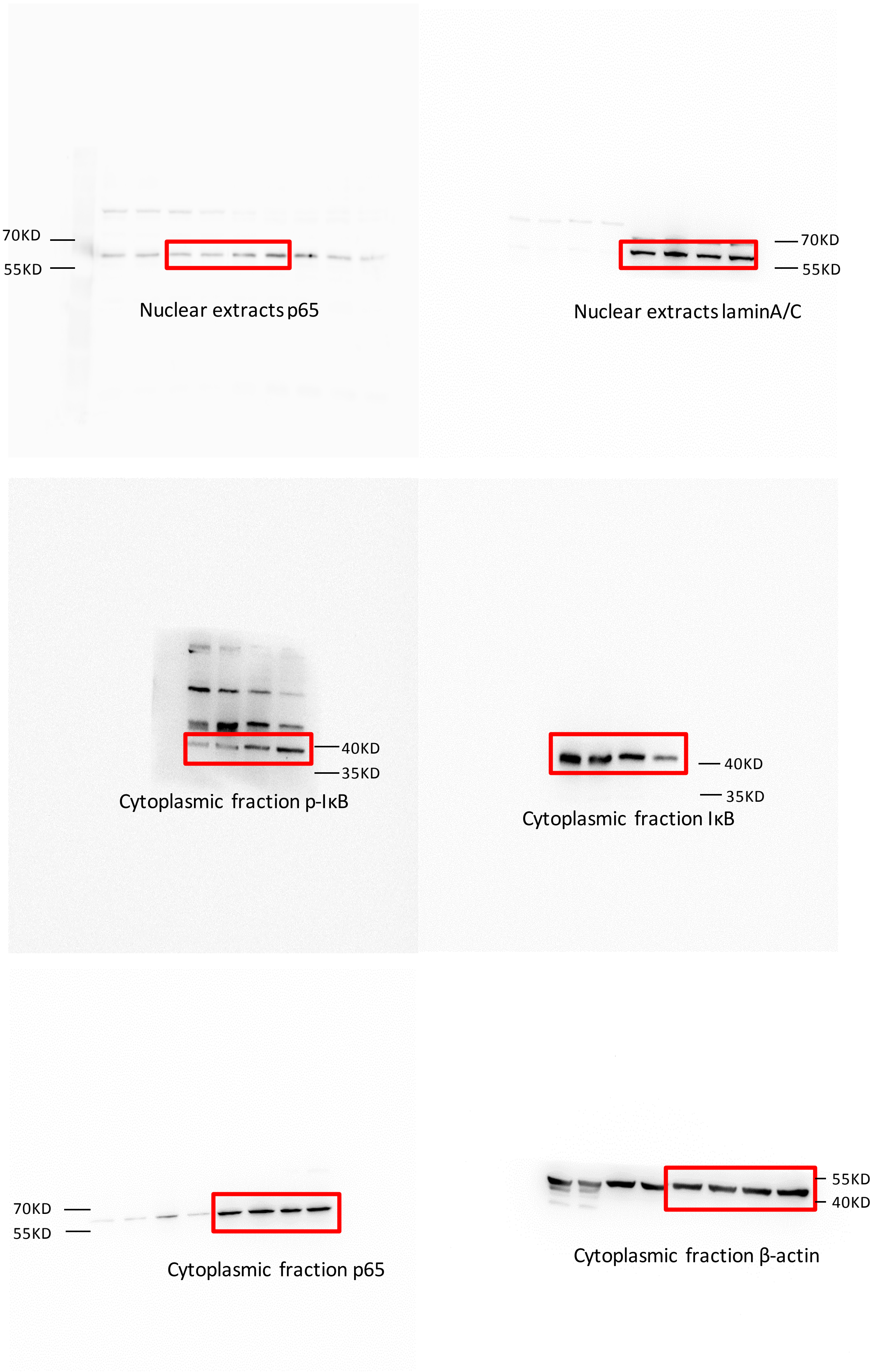

Supplement: Supplementary file 1 — Supplementary Information [file 41598_2018_27302_MOESM1_ESM.pdf]
